# The viral origin of myalgic encephalomyelitis/chronic fatigue syndrome

**DOI:** 10.1371/journal.ppat.1011523

**Published:** 2023-08-17

**Authors:** Maureen R. Hanson

**Affiliations:** Department of Molecular Biology and Genetics, Cornell University, Ithaca, New York, United States of America; University of Iowa, UNITED STATES

ME/CFS is a disabling and often severe disease, so-far incurable, that has long been associated with discrete outbreaks and sporadic incidents of viral-like illness. First, a word about the controversial name. The designation “Myalgic Encephalomyelitis” (abbreviated ME) originated following an outbreak at London’s Royal Free Hospital in 1955. More than 200 members of the hospital staff became disabled [1]. Melvin Ramsay, MD, eventually published important case descriptions in *Lancet* [2]. He coined “ME” based on predominant symptoms of muscle pain (myalgia) and effects on the brain (encephalo), spinal cord (myel), and inflammation (itis). For 32 years, “ME” was deemed acceptable until, in 1987, the Centers for Disease Control (CDC) convened an extramural committee to change the name. CDC did so in response to a series of outbreaks of a similar, if not identical, illness in the United States, introducing “chronic fatigue syndrome” in 1988 [3].

Because the CDC name trivializes the serious nature of the disease, the patient community and many medical professionals prefer ME, which continues to be widely used in the United Kingdom and Europe. In 2015, a US Institute of Medicine (IOM) committee recommended yet another name, Systemic Exertion Intolerance Disease [4], which has been largely ignored. Should inflammation of the brain and spinal cord be definitively shown with modern methods, the name Myalgic Encephalomyelitis will finally be vindicated. The compromise name ME/CFS is now used most frequently and will be used here despite its faults.

## Can any infection lead to ME/CFS?

There is actually no proof that multiple different pathogens can cause ME/CFS. Yet, this hypothesis persists largely due to the overinterpretation of data from at least 2 studies. The limited evidence depends on how ME/CFS is defined.

**Fig 1:**
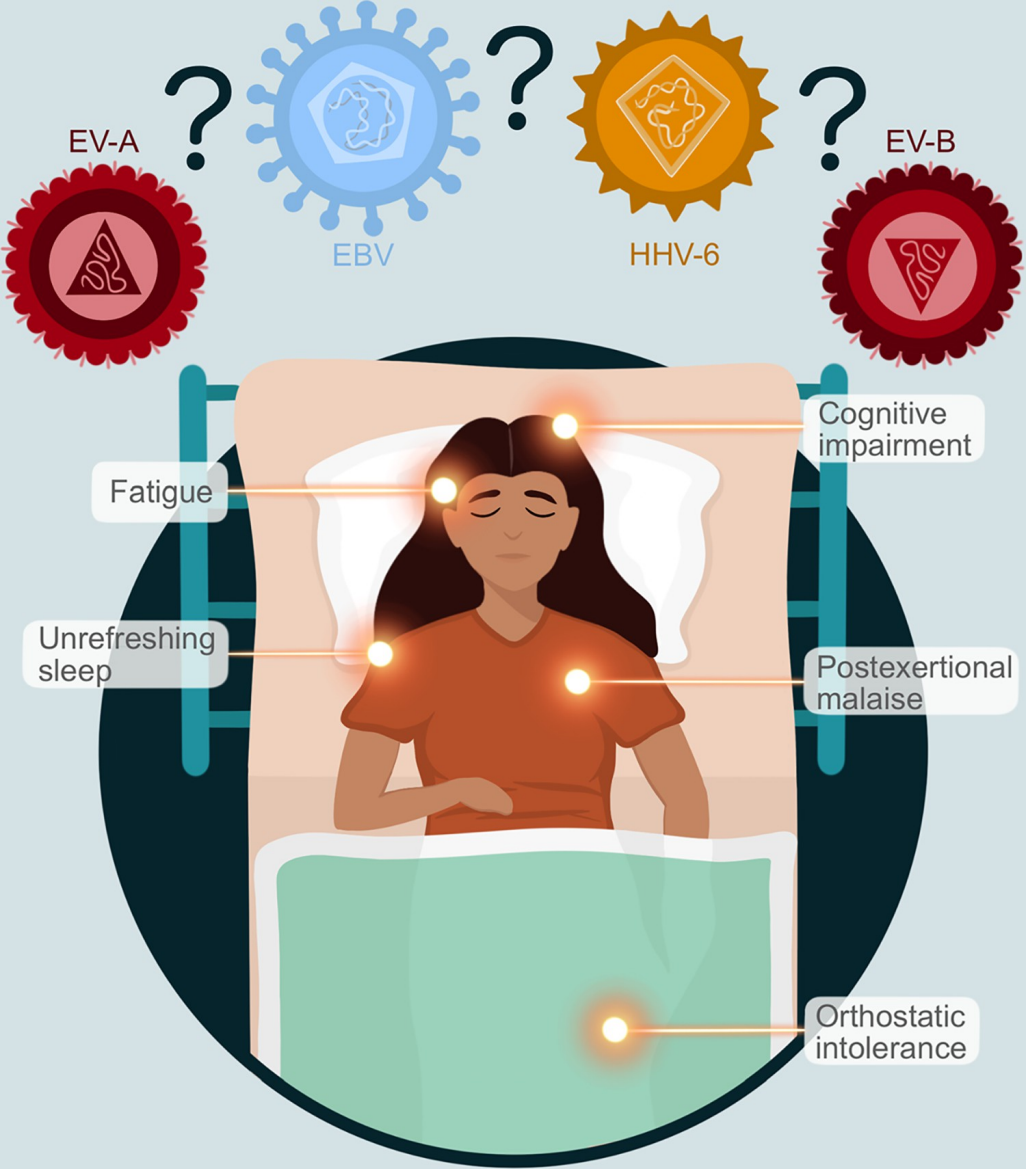
The 5 diagnostic criteria for ME/CFS recommended by the IOM committee. Artist credit: Jessica Maya.

Currently, research groups and clinicians typically use any one of 3 definitions: the so-called Fukuda criteria (CDC’s 1988 criteria) [3], the Canadian Consensus Criteria of 2003 [4], and criteria suggested by the IOM committee [5]. Of these, the Fukuda criteria are considered somewhat obsolete, as there is no requirement for core symptoms [6]. The 2015 IOM Committee required (1) impaired ability to engage in pre-illness activity levels that lasts for more than 6 months; (2) increased symptoms following physical, mental, or emotional exertion that would have been tolerated pre-illness (post-exertional malaise); and (3) unrefreshing sleep. For diagnosis, an individual must also exhibit either cognitive impairment and/or orthostatic intolerance [5] (Fig 1).

A 2006 Australian study is oft cited as proof that virtually any number of infections cause ME/CFS. Researchers followed 253 people in Dubbo for 12 months. Of these, 171 had verified acute Epstein–Barr virus infection, Q fever (caused by *Coxiella burnetii*), or Ross River virus infections, while 82 were suspected of having one of these infections. Nine percent of the participants exhibited what was termed “post-infective fatigue syndrome,” encompassing 6 symptoms, of which only 2 (fatigue and neurocognitive difficulties) are also found in the IOM criteria. Whether all of these individuals have PEM or unrefreshing sleep was not reported. Likely few of them would fulfill the more recent criteria.

A recent study of medical claims data in the US produced an ME/CFS prevalence of 1.7 to 3.4 million [7]. Using this information as well as data from other studies, we estimated the pre-2020 worldwide prevalence of ME/CFS to be around 67 million [8]. If, for the sake of argument, we assume that any of the Dubbo participants did have ME/CFS by all criteria, it would be highly unlikely for Ross River virus, which is endemic in Australia and Papua New Guinea, and Q fever, mainly a disease of people highly exposed to farm animals, to account for more than a minute fraction of cases of ME/CFS. In the US, usually fewer than 200 people a year are reported with Q fever (https://www.cdc.gov/qfever/stats/index.html).

In another study, this one from Norway, investigators cited influenza as an instigator of ME/CFS. The researchers evaluated the risk of acquiring ME/CFS during a 2009 influenza A (H1N1) pandemic. They concluded that influenza infection was associated with a more than 2-fold increased risk of ME/CFS during the subsequent 3 years compared to people who received a vaccination but did not become ill [9]. Some claim this study as proof that influenza A can cause ME/CFS. However, investigators included people who were ill with a flu-like illness but failed to verify that the malady was actually influenza A. If someone received a medical record code for H1N1 infection during the height of the pandemic (October 1 to December 31, 2009), it was assumed to be influenza A even if not confirmed. While 2.4% of the population were given a diagnosis of H1N1 based on symptoms during that period, only 0.3% of the population had the identity of the virus actually certified in a laboratory [9]. A more robust conclusion is that people who were vaccinated, but did not experience a flu-like illness, had only half the risk of ME/CFS compared to individuals who had a flu-like infection, vaccinated or not.

## Why is the enterovirus family the most likely culprit in ME/CFS?

History offers persuasive evidence to suspect the enterovirus (EV) family of causing ME/CFS.

Both circumstantial and direct evidence exists to support such a conclusion [10,11]. For one, early outbreaks of ME/CFS often coincided with outbreaks of polio, which is caused by 3 members of the enterovirus C family. Typically, ME/CFS victims lived or worked in close or intimate proximity to polio patients in well-documented cluster outbreaks [12,13]. Given that enteroviruses can be spread through contaminated water and food, a possible explanation for the coincident outbreaks could be the simultaneous circulation of multiple enteroviruses transmitted in the same ways, as well as possible recombination between enteroviruses to create one with unusual properties.

Moreover, ME/CFS case descriptions by health professionals of the era are consistent with the features of known enteroviral infections. Nevertheless, anyone who reads these accounts will be struck by some diversity between the symptom clusters and even their severity in different outbreaks [1,12–15]. This variability is readily explained, however, if different members of the large EV family, or different variants of members of the same EV clade, were responsible for the outbreaks. Tremendous sequence diversity characterizes the *Enterovirus* genus, even among members of the same clade [16].

Aside from associations of EVs with past ME/CFS outbreaks and sporadic cases, well documented by Dr. Byron Hyde [12,13], there is also more contemporary evidence that favors enteroviruses [12,13]. Infectious disease specialist Dr. John Chia and colleagues have provided compelling data on behalf of enteroviruses in the form of case histories and experimental data demonstrating chronic viral infection.

For instance, 3 ME/CFS patients with definite acute EV infections confirmed by RNA analysis at onset were followed longitudinally. Stomach biopsies were positive for EV infection years after onset [17]. When Chia and colleagues analyzed many more such biopsies for EV VP1 and RNA, ME/CFS patients had a much higher proportion of positive signals compared to controls [18]. Subsequently, Chia demonstrated that extracts of stomach biopsies from people with ME/CFS were able to infect immunodeficient mice [19]. Unfortunately, the complete RNA genomes of EVs in these biopsies were not obtained. If such samples become available today, they would be highly informative. In addition to stomach biopsies, other tissue samples—for example, heart, brain, muscle—would be particularly valuable for screening for chronic enteroviral infection, while blood samples are unlikely to be informative once a few weeks have passed since the acute infection [11]. The absence of available cadaver samples from ME/CFS cases handicaps thorough investigations of tissues and organs for the presence of chronic viral infection.

A vast number of enteroviral infections occur worldwide every year [16], with reports that as many as 50% are asymptomatic [20]. Approximately one-third of ME/CFS patients cannot trace their onset to a flu-like illness. Quite possibly, they could have had an asymptomatic case or a mild one long before the onset of the characteristic ME/CFS symptoms. As a result, they may fail to ascribe their illness to a virus. Instead, they may point to some other life stress—physical or emotional. Stressful life events could play a role in exacerbating a viral infection and stress is known to affect the immune system [21].

One reason EVs are often dismissed as causative factors may be explained by the high frequency with which enteroviral infections are experienced throughout the world [22]. Serological studies have not detected differential exposure to a virus in ME/CFS patients versus controls. Most patients who are recruited for studies have been ill for many years and they as well as controls will exhibit antibodies to multiple enteroviruses from past infections upon serological testing, preventing discrimination among cohorts. The strongest hypothesis for the cause of ME/CFS outbreaks is spread of an uncommon variant of a common virus family. A variant could also be responsible for sporadic cases. Conversely, the sporadic cases may reflect an uncommon reaction to common viruses, perhaps due to genetic or other biological factors such as coinfections and comorbidities.

A new localized outbreak would offer an ideal opportunity to pinpoint a pathogen that could have caused pre-2020 ME/CFS cases. With one exception, the CDC dismissed scores of outbreaks reported to the agency in the 1980s. At the time, these reports were routinely denigrated as hysterical illness [15]. Even the less-sophisticated technology of the era might have identified viral sequences [23] as well as serological evidence for the presence of circulating enteroviruses. Certainly, few if any ME/CFS outbreaks have been reported during the 25 years prior to 2020. Given our estimate of 67 million cases worldwide [8], it is reasonable to assume many hundreds of thousands of cases or more have occurred during that time. It makes sense that both more sporadic cases and outbreaks that are not due to coronaviruses will arise in the future. Dismissing future outbreaks of ME/CFS as psychological will be more difficult now that post-acute SARS-CoV-2 illness has been recognized, widely publicized, and subjected to extensive study [24,25].

## What is the relationship between human herpesviruses (HHVs) and ME/CFS?

A striking number of ME/CFS patients mention an acute infection with EBV or some other human herpesvirus (HHV) as the start of their illness. Whether this is true or not is not known. If someone has a long course of mononucleosis, an additional virus that may or may not cause symptoms might be necessary for induction of ME/CFS. Consider an example: in one study, 13% of adolescents with diagnosed cases of infectious mononucleosis fit ME/CFS diagnostic criteria 6 months later. Twenty-four months after diagnosis, the percentage decreased to 4% [25]. Three possibilities exist: (1) EBV or another HHV can directly, on its own, cause ME/CFS; (2) an acute HHV infection makes an individual susceptible to ME/CFS when a subsequent viral infection occurs; (3) even when an HHV has not induced ME/CFS, its reactivation helps perpetuate the illness. The latter 2 mechanisms could even coexist. Also, the third possibility may describe a subset of patients who improve following treatment with anti-herpesviral drugs [26,27].

Infections with HHVs are common and lifelong. Healthy people maintain viruses such as EBV in a latent state. But herpesviruses commonly reactivate under a variety of stressful conditions or illnesses such as ME/CFS or acute SARS-CoV-2 [28,29]. Following the mid-1980s ME/CFS outbreak in Incline Village, NV, a detailed study documented active replication of the newly discovered HHV-6 in blood [30]. Because most people are infected early in life, new HHV infection would be inconsistent with the epidemiology of the outbreak. Reactivation of dormant HHVs as a result of onset of ME/CFS may have sometimes been mistaken for a new infection, resulting in patients believing that an HHV induced their chronic illness.

## Should the post-SARS-CoV-2 infection syndromes be called “ME/CFS”?

The US government devised the name Post-Acute Sequalae of COVID-19 (PASC) to describe a post-acute illness syndrome suffered by victims who endured this deadly virus. PASC patients’ symptoms include observable damage to the heart, kidney, lungs or other organs, lung damage from invasive mechanical ventilation, blood clots, rashes, tinnitus, disturbances of taste and smell, as well as a plethora of other symptoms [25,31]. Most relevant to ME/CFS is the fact that some people who suffered mild or asymptomatic cases of COVID-19 later began experiencing a post-viral illness that fulfills most or even all of the IOM criteria for ME/CFS [32]. Increasingly, these people are then told by their doctors that they have ME/CFS, but the IOM diagnostic criteria were created 6 years before SARS-CoV-2 emerged. The IOM committee could not have possibly considered symptoms characteristic of PASC. Thus, while it would be correct to say that someone has post-COVID illness with symptoms diagnostic of ME/CFS, referring to a post-COVID syndrome as actual ME/CFS will confuse the scientific literature and cloud clinical trials. Careful categorizations of the many forms of post-COVID illness are only just beginning [33,34]. Recently, a study of 9,764 individuals experiencing symptoms following acute COVID-19 resulted in classification of the cases into 4 subgroups and the identification of 12 core symptoms among 44 that were considered [35]. Unfortunately, one of the key ME/CFS symptoms—unrefreshing sleep—was not evaluated. The 12 core symptoms include ones that are not identified as core symptoms in any of the ME/CFS diagnostic criteria. Nevertheless, the intriguing overlap in symptoms between some forms of post-COVID illness and ME/CFS suggests that disruptions in the same pathways may be occurring in both diseases, but to conclude the 2 syndromes are identical without more data, especially at the molecular level, is currently unwarranted.

Just one example: data is emerging to suggest some who are suffering long-term symptoms may have a chronic SARS-CoV-2 infection [36,37]. But no one who acquired ME/CFS before 2020 became ill as a result of SARS-CoV-2. Any SARS-specific antiviral treatments will not be effective for ME/CFS patients. Nevertheless, the fact that there are common symptoms means that interventions to ameliorate those symptoms may be valuable in both illnesses [25]. Given that the present estimate of 65 million long COVID sufferers worldwide [25] is nearly identical to our estimate of ME/CFS sufferers worldwide [8], the latter of whom have been ill for years and even decades longer, suggests that both diseases, not only PASC, deserve major investments of money and time from the world’s research communities.

## Conclusion

Ignoring the abundant evidence for EV involvement in ME/CFS has slowed research into the possible dire but hidden consequences of EV infections, including persistence in virus reservoirs. Prior to the SARS-CoV-2 pandemic, the ability of RNA viruses to persist in tissues for long periods was largely ignored. Further, recognizing that EVs are prime candidates for causing ME/CFS suggests how critical it is to pursue a relevant inquiry into this diverse virus family. Do hidden reservoirs harbor these viruses? Have they induced autoimmunity through molecular mimicry? Is it past or current infection that has resulted in the many findings of immune dysfunction in ME/CFS?
